# Effect of Health Education Programme on the Knowledge of and Attitude about Sickle Cell Anaemia among Male Secondary School Students in the Jazan Region of Saudi Arabia: Health Policy Implications

**DOI:** 10.1155/2019/9653092

**Published:** 2019-07-25

**Authors:** Mohammed Mahmoud Kotb, Mohammed J. Almalki, Yasser Hassan, Anwar Al Sharif, Maseer Khan, Kamaludin Sheikh

**Affiliations:** ^1^Faculty of Public Health & Tropical Medicine, Jazan University, Saudi Arabia; ^2^Faculty of Medicine, Department of Community Medicine, Ain Shams University, Egypt

## Abstract

This study was conducted to develop an instructional programme on sickle cell anaemia (SCA) and test the effect of the programme on the secondary school students' knowledge of and attitude towards sickle cell anaemia in the Jazan region of Saudi Arabia. A pretest/posttest one-arm interventional study was conducted at the Faculty of Public Health and Tropical Medicine, Jazan University, with a convenience sample of 120 male students. The intervention consisted of two interactive sessions about sickle cell anaemia and premarital screening. The mean student knowledge score was 6.04 ± 3.02 on the pretest, which improved to 10.73 ± 3.47 on the posttest, with a statistically significant difference (t = 15.2, p < 0.001). There was no significant difference in the responses pertaining to attitude before and after the health education intervention. The policy implications of these findings are discussed to improve the performance of the Saudi healthcare system in dealing with this costly inherited disease.

## 1. Introduction

Sickle cell anaemia (SCA) is a recessive single-gene inherited disorder. The disease is characterized by being difficult to treat and causing significant morbidity and mortality that pose financial burdens at the family and country levels. It is one of the most important single-gene disorders in human beings [1]. The main characteristics of sickle cell anaemia are severe anaemia that might necessitate blood transfusion, repeated chest infections, episodes of pain, leg ulcers, pulmonary hypertension, splenic infarction, repeated strokes, necrosis of joints, and renal failure [1]. Sickle cell anaemia patients usually require repeated hospitalization due to the associated complications, and many of the affected children suffer from premature death. Approximately 50% of surviving patients have documented irreversible organ damage. The risk of early death is highest among patients who have had severe complications, such as recurrent acute chest syndrome, renal failure, and pulmonary hypertension [2–4].

The prevalence rate of sickle cell anaemia in Saudi Arabia was estimated to be 24 per 10,000. The regional distribution showed that the highest prevalence rates are found in the eastern and southern regions [5]. The Saudi Ministry of Health (MOH) started a premarital national screening programme for SCA in 2004. The main objective of this programme was to reduce the prevalence of sickle-cell anaemia in the population by reducing the number of at-risk marriages [6]. According to the programme guidelines, couples seeking to become engaged have to visit the nearest primary health care clinic to apply for blood sample analysis and a premarital certificate [6]. This initiative may reduce the burden of disease at both the community and health care system levels.

Previous studies in Saudi Arabia have documented poor awareness in the population about the hereditary transmission of sickle cell anaemia, the associated complications, and the premarital screening services [6]. Many of the new couples do not have the necessary information related to marriage, reproductive health and family planning [7]. No studies have addressed the role of educational interventions in improving the level of knowledge and attitude of the targeted population groups.

Improving the peoples' level of knowledge and attitudes will certainly improve their understanding and cooperation and may decrease the number of marriages between carriers. It is important to assess and improve the level of knowledge and attitude in young people because they are future parents of society [7].

Health education could provide individuals with the necessary knowledge, skills, and motivation to make healthier lifestyle choices [7]. Likewise, preventive health education programmes can help reduce financial expenditures on treatment and curative practices. Health educational programmes targeting high school students in Jamaica were effective at increasing awareness of genetic blood diseases and premarital screening services [8]. Health educational programmes are expected to improve the outcome of the premarital screening programme by raising awareness and understanding of the hereditary nature of sickle cell anaemia and its associated complications. Therefore, the primary aim of this study was to develop an instructional programme on sickle cell anaemia for secondary school students and pilot test its effects on the levels of students' knowledge and attitude related to SCA and premarital screening services in the Jazan region, where the rate of sickle cell anaemia is extremely high. In addition, this study provides recommendations for the healthcare system to facilitate a reduction in the financial burdens of this critical disease.

## 2. Subjects and Methodology

### 2.1. Design, Sample, and Procedures

This interventional study was conducted by the Faculty of Public Health and Tropical Medicine, Jazan University, Saudi Arabia, during the academic year 2017-2018. The study was conducted in the Jazan region located in southern Saudi Arabia, where SCA is endemic. It involved a convenience sample of 120 students from the thirteen male secondary schools that agreed to participate in the study. Out of the total schools, four schools were located in rural areas, and the rest of the schools (nine schools) were located in urban areas. Of all the students, 70 were from rural areas and 50 were city dwellers.

After obtaining verbal consent from the students to participate in the study, students were given an overview of the purpose of the study and the different sections and questions of the study questionnaire. Then, the students were provided with the questionnaire and asked to fill out the form to assess their baseline knowledge of and attitude towards sickle cell anaemia and premarital screening. It took an average of 20 minutes for students to complete the study questionnaire. After the baseline assessment, the health education intervention was conducted. It involved two interactive sessions using audiovisual aids. After the completion of the health education sessions, re-assessment of the students' knowledge and attitude was performed with the same questionnaire used in the baseline assessment.

### 2.2. The Instructional Health Education Programme

A focus group discussion session was conducted with 20 secondary school students. The focus group session was administered by a moderator who acted as a facilitator and a note taker who was responsible for recording the proceedings of the session. The findings of the focus group discussion session were analysed to identify the level of awareness and the prevailing misconceptions and beliefs related to sickle cell anaemia and premarital screening services. The materials for the health education intervention were prepared based on the results of the focus group sessions, informal meetings with practising paediatricians and a review of the relevant literature, both locally and internationally.

The content of the first health education session mainly focused on the following points: sickle cell anaemia as a hereditary blood disease and its socio-medical burden, including the pathophysiology and the potential complications, as well as the concept of case and carrier. The second health education session covered the following topics: mode of disease inheritance from parents to children, role of consanguinity in perpetuating disease occurrence, the importance of premarital investigation, and the concept of fit and unfit marriages. Each of the two sessions continued for approximately 45 minutes and was followed by a group discussion. At the end of the second lecture, a booklet about sickle cell disease was given to each student to share the given information with their family members.

### 2.3. Instrument

A prevalidated self-administered questionnaire was used to assess the effect of the health education sessions on the students' knowledge of and attitudes towards sickle cell anaemia. The questionnaire included three sections. The questions in the first section covered the sociodemographic profile, and the questions in the second and third sections focused on the students' knowledge and attitude, respectively. Thirteen questions were used to assess the overall knowledge of the students of sickle cell anaemia and premarital screening. Each correct answer in the knowledge domain received a mark of 1, while wrong or “do not know” answers received a mark of 0. This gave a total score ranging from 0 to 13.

The attitude of the students was assessed through four statements based on a five-point Likert scale, with the options of strongly agree, agree, neutral (undecided), disagree, and strongly disagree. The four statements in the attitude domain pertained to the support of consanguineous marriage, the support of premarital screening, the right to proceed with high-risk marriages, and the support of a legal ban on high-risk marriages. The questionnaire was reviewed and approved by a team of two health education experts. The purpose of this content validation was to ensure that the questions were properly formulated and the content was appropriate. Based on the feedback of the two experts, the necessary modifications were made with regard to the structure and arrangement of the knowledge questions and attitude statements.

### 2.4. Data Analysis

Data were entered into and analysed with SPSS version 16.0. Both descriptive and inferential statistics with paired samples t-tests and Wilcoxon signed-rank tests were conducted. A paired samples t-test was used to test the mean difference between pretest and post-test knowledge scores. The Wilcoxon signed-rank test was used to test the difference between pretest and posttest scores on the attitude statements.

## 3. Results

A total of 129 questionnaires were distributed and collected at both the baseline survey and posttest assessment. Nine questionnaires were excluded due to incomplete data.

Table 1 shows the sociodemographic profile of the students who participated in the study. As shown in Table 1, 58.3% of the studied students resided in villages, and the rest (41.7%) lived in cities. The percentage of those who had sickle cell anaemia was 6.7%. With regard to the parents' education, 29.1% of the fathers of the respondents primary education or less, while 42.4% had university level educations. With regard to the mothers' education, 15.8% had less than primary school level education, while 30.8% had university level education.

Table 2 shows the students' performance on the knowledge domain. The lowest baseline scores were obtained for questions 6 (presence of symptoms in SCA carriers), 9 (marriage between a couple with one carrier and one healthy individual is considered risky), 12 (availability of a complete cure for SCA), and 13 (the possibility of converting an SCA carrier to an SCA patient), and they were 10.8%, 16.7%, 12.5%, and 10.8%, respectively. There was a marked increase in the level of students' knowledge on all knowledge items from the pretest to the posttest.

Table 3 shows that the mean baseline knowledge score was 6.04 (SD 3.02), which increased to 10.73 (SD 3.47) after the health education programme. The mean difference (4.70) was statistically significant (95% CI 4.086, 5.307). These findings indicate that there was a significant increase in the mean knowledge score of 78% compared to the baseline. The increase was calculated using the following formula: (pretest mean score-post-test mean score) / pre-test mean score.

Table 4 shows the students' responses to the attitude statements at baseline and after the intervention. The nonparametric Wilcoxon-signed rank test indicated that the difference between the baseline and postintervention responses on the attitude statements (support consanguinity, support premarriage screening, proceed with risky marriage, and support banning risky marriage by law) were not statistically significant (p > 0.05).

## 4. Discussion

This study was conducted to develop an instructional programme about SCA and pilot test its effects on the knowledge of and attitude towards SCA of secondary school students in the Jazan region of Saudi Arabia. The baseline assessment revealed gaps in the knowledge of the students, and the postintervention assessment showed significant improvement in the knowledge scores. The majority of the students were unaware of the basic information related to SCA, such as it being a hereditary disease, that a carrier does not suffer from symptoms, the role of consanguineous marriage in perpetuating the problem, the incurability of SCA and the pattern of disease inheritance. Nearly 90% of the participants (Table 2) thought that sickle cell anaemia carriers could become sickle cell anaemia patients. This finding gives an indication of the need for more efforts to reduce the expected effects of this genetic disease through public awareness activities and to control the increasing pressure on health services.

In countries such as Nigeria, where SCA is a public health problem, educational activities are conducted in social studies classes in secondary schools to increase awareness and sensitize students to the problem [8, 9]. However, in Saudi Arabia, although there are low levels of awareness and high prevalence rates of SCA, it is still not a part of the school curriculum. Therefore, there is a pressing need to introduce topics related to SCA into both science and social studies classes in schools [10].

Population-based premarital genetic screening coupled with health education and counselling services should be the main focus of efforts to control SCA in countries with high prevalence rates, such as Saudi Arabia. For that reason, the Saudi Ministry of Health started a premarital screening programme with mandatory testing for sickle cell anaemia in 2005. However, the programme focused mainly on premarital screening and individual counselling services and neglected population-based health education activities. It is very difficult to control genetic disorders if the community awareness levels are low.

The health education programme significantly improved the overall level of students' knowledge of sickle cell anaemia and premarital screening (Table 3). This is in line with the findings of a previous study carried out in Jeddah, Saudi Arabia, that found that health education interventions could significantly raise the level of the students' knowledge related to SCA [11].

The students' attitudes seem to be ambivalent as the most common student responses to different attitude statements tended to be neutral (“undecided”) for both baseline and postintervention surveys (Table 4), with the exception of the statement related to “support pre- marital screening” to which the predominant response was to strongly support screening. Similar results were noted by Olatona et al. [12] in Nigeria, where it was found that respondents had ambivalent attitudes towards prenatal screening for sickle cell anaemia. Such variations in the attitude domain between different health education interventional studies could be attributed to a multiplicity of variables, including the effectiveness of health education, perceived seriousness of the disease, perceived barriers in the community that could restrain the person from making positive changes, perceived benefits of a positive attitude, and perceived susceptibility to disease [13]. Based on the health belief model, achieving a high level of knowledge of the disease is not a guarantee for achieving attitudinal changes, as the implementation of the gained knowledge necessitates the existence of perceived personal susceptibility, perceived seriousness of the disease, and lack of cultural habits that could hinder attitudinal changes [14]. Within the Saudi society, there is an embedded cultural practice of marrying a cousin to sustain a strong connection to the tribe [15] as well as to preserve the traditions and wealth of the family [16]. Accordingly, the arrangements for marriages are predetermined and are not considered negotiable, irrespective of the potential consequences [16]. Within this context, we would expect the students to have an ambivalent attitude, faced with the actual societal practices on the one hand and the information provided during health education on the other hand. Other explanations reported by previous studies for unchanged attitudes, despite participants' increased understanding of the severity of the disease and the benefits of sickle cell anaemia screening, are the individual believing that the chance of having a new-born with sickle cell anaemia in the family is low [17], that sickle cell testing is painful [18], and that the genetic information may be misused [19]. It seems that there is a need to emphasize the sensitization of students to the susceptibility and the seriousness of the disease and to adopt a community-based health education strategy to change the practice of consanguineous marriage. There is also a need to integrate the topic of sickle cell anaemia into the school curriculum as a social problem with devastating health consequences.

In summary, the proposed health education intervention increased the overall knowledge of the students; however, the attitude of the participants remained unchanged. There is a need for frequent health education campaigns to sensitize the students and increase their understanding of the seriousness of the disease and the hazards of consanguineous marriage. This should form a part of a comprehensive community-based health education programme to overcome the deeply rooted cultural habits and practices. Such efforts and programmes may also contribute to reducing the costs, financial burdens, and economic impacts of the disease.

The following are the study limitations: the study sample was restricted to the male students, and a nonprobability sampling approach was used. Another limitation was the lack of a comparison group and followup to determine changes in knowledge and attitudes over time. Therefore, a better study design could have been adopted based on random sampling with a comparison group and postintervention repeated assessments.

## Figures and Tables

**Table 1:** General characteristics of the students (n = 120).

Variables	No.	%
Place of residence		
Village	70	58.3
City	50	41.7
Do you have SCA?		
Yes	8	6.7
No	112	93.3
Father's education level		
No education	4	3.3
Primary	31	25.8
Secondary	34	28.4
University	51	42.5
Mother's education level		
No education	19	15.8
Primary	38	31.7
Secondary	26	21.7
University	37	30.8

**Table 2:** Baseline and postintervention correct answers on knowledge questions.

No.	Knowledge question / correct answer*∗*	Before	After
		Number (%)	Number (%)
1	Is SCA common in Jazan? Yes	46 (38.3%)	88 (73.3%)

2	Is SCA a hereditary disease? Yes	75 (62.5%)	104 (86.7%)

3	Is SCA an infectious disease? No	68 (56.7%)	92 (76.7%)

4	Which type of blood component is affected by SCA? Red blood cell	59 (49.2%)	100 (83.3%)

5	Does consanguinity increase the probability of SCA? Yes	53 (44.2%)	84 (70.0%)

6	Does an SCA carrier suffer from symptoms? No	13 (10.8%)	63 (52.5%)

7	Is a marriage between two diseased persons risky? Yes	88 (73.3%)	102 (85.0%)

8	Is a marriage between two carriers risky? Yes	66 (55.0%)	82 (68.3%)

9	Is a marriage between one carrier and one healthy individual risky? No	20 (16.7%)	51 (42.5%)

10	Should an SCA patient abstain from marriage so as not to have diseased children? No	53 (44.2%)	76 (63.3%)

11	Should an SCA carrier abstain from marriage so as not to have diseased children? No	61 (50.8%)	84 (70.0%)

12	Is there a complete cure for SCA? No	15 (12.5%)	94 (78.3%)

13	Can an SCA carrier become an SCA patient? No	13 (10.8%)	85 (70.8%)

*∗* Correct answer.

**Table 3:** Baseline and postintervention knowledge scores.

Knowledge score	Mean (SD)	95% CI of the mean diff.		Paired t-test (df)	P-Value
		Lower	Upper		
Baseline	6.04 (3.02)	4.70 (4.086, 5.307)		15.236 (119)	< 0.001
Post-intervention	10.73(3.47)				

**Table 4:** Baseline and post-intervention answers to attitude questions.

Attitude parameter		Strongly Agree	Agree	Undecided	Disagree	Strongly Disagree	Total	P value
Support consanguineous marriage	Pre-test	9 (7.5%)	19 (15.8%)	64 (53.3%)	18 (15.0%)	10 (8.3%)	100%	0.72
	Post-test	14 (11.7%)	23 (19.2%)	44 (36.7%)	19 (15.8%)	20 (16.7%)	100%	

Support pre-marriage screening	Pre-test	71 (59.2%)	31 (25.8%)	13 (10.8%)	3 (2.5%)	2 (1.7%)	100%	0.48
	Post-test	84 (70.0%)	17 (14.2%)	13 (10.8%)	3 (2.5%)	3 (2.5%)	100%	

Proceed with risky marriage	Pre-test	5 (4.2%)	22 (18.3%)	35 (29.2%)	45 (37.5%)	13 (10.8%)	100%	0.88
	Post-test	14 (11.7%)	20 (16.7%)	27 (22.5%)	31 (25.8%)	28 (23.3%)	100%	

Support banning risky marriage by law	Pre-test	19 (15.8%)	17 (14.2%)	42 (35.0%)	26 (21.7%)	16 (13.3%)	100%	0.30
	Post-test	14 (11.7%)	17 (14.2%)	49 (40.8%)	18 (15.0%)	22 (18.3%)	100%	

*∗*Wilcoxon signed-rank test

## Data Availability

The data used to support the findings of this study are available from the corresponding author upon reasonable request.
